# Association of State Opioid Duration Limits With Postoperative Opioid Prescribing

**DOI:** 10.1001/jamanetworkopen.2019.18361

**Published:** 2019-12-27

**Authors:** Sunil Agarwal, John D. Bryan, Hsou Mei Hu, Jay S. Lee, Kao-Ping Chua, Rebecca L. Haffajee, Chad M. Brummett, Michael J. Englesbe, Jennifer F. Waljee

**Affiliations:** 1 Department of Anesthesiology, Emory University, Atlanta, Georgia; 2 Michigan Opioid Prescribing Engagement Network, Ann Arbor; 3 Gerald R. Ford School of Public Policy, University of Michigan, Ann Arbor; 4 Department of Surgery, Michigan Medicine, Ann Arbor; 5 Susan B. Meister Child Health Evaluation and Research Center, Department of Pediatrics, University of Michigan Medical School, Ann Arbor; 6 Department of Health Management and Policy, University of Michigan School of Public Health, Ann Arbor; 7 Department of Anesthesiology, Michigan Medicine, Ann Arbor

## Abstract

**Importance:**

Since the Centers for Disease Control and Prevention published opioid prescribing guidelines in March 2016, 31 states have implemented legislation to restrict the duration of opioid prescriptions for acute pain. However, the association of these policies with the amount of opioid prescribed following surgery remains unknown.

**Objective:**

To examine the association of opioid prescribing duration limits with postoperative opioid prescribing in Massachusetts and Connecticut, the first 2 states to implement limits after March 2016.

**Design, Setting, and Participants:**

This interrupted time series analysis and cross-sectional study examined immediate level and slope changes in monthly outcomes after prescribing limit implementation in Massachusetts and Connecticut. These states implemented 7-day limits on initial opioid prescriptions on March 14, 2016, and July 1, 2016, respectively. Using the 2014 to 2017 IBM MarketScan Research Database, 16 281 opioid-naive adults in these states who filled a prescription within 3 days of surgery between July 1, 2014, and November 30, 2017, were identified. Data were analyzed from December 2018 to June 2019.

**Main Outcomes and Measures:**

The primary outcome was the prescription size in oral morphine equivalents (OMEs) for the initial postoperative opioid prescription (one 5/325 mg hydrocodone-acetaminophen pill = 5 OMEs). Secondary outcomes included days supplied in the initial prescription and the proportion of initial prescriptions exceeding a 7-day supply.

**Results:**

In total, 16 281 opioid-naive patients (9708 [59.6%] female; median [interquartile range] age range, 45-54 [35-44 to 55-64] years) undergoing surgical procedures were included. In Massachusetts, there were 5340 and 5435 patients in the preimplementation and postimplementation periods, respectively. In Connecticut, there were 2869 and 2637 patients in the preimplementation and postimplementation periods, respectively. Limit implementation in Massachusetts was associated with an immediate mean level decrease in prescription size (−38 OMEs [95% CI, −44 to −32 OMEs]) and with a mean decrease in slope (−1.5 OMEs/mo [95% CI, −2.1 to −0.9 OMEs/mo]). Implementation was also associated with an immediate mean level decrease in days supplied (−0.4 days [95% CI, −0.6 to −0.2 days]) and the proportion of prescriptions exceeding a 7-day supply (−5.9 percentage points [95% CI, −7.9 to −3.9 percentage points]). In contrast, limit implementation in Connecticut was not associated with level or slope changes in any outcome.

**Conclusions and Relevance:**

Opioid prescribing duration limits had a variable association with postoperative opioid prescribing in Massachusetts and Connecticut. The mean opioid prescription size filled, days supplied, and prescribing exceeding a 7-day supply decreased after limit implementation in Massachusetts only. Given the potential differences in policy dissemination and uptake, efforts to reduce opioid prescribing should also include surgeon education and evidence-based prescribing recommendations.

## Key Points

**Question:**

What is the association of opioid prescribing duration limits with postoperative opioid prescribing in Massachusetts and Connecticut?

**Findings:**

This interrupted time series analysis and cross-sectional study of 16 281 opioid-naive patients found that limit implementation in Massachusetts was associated with an immediate mean level decrease of 38 oral morphine equivalents in prescription size and with a mean decrease in slope of 1.5 oral morphine equivalents/mo. In contrast, limit implementation in Connecticut was not associated with level or slope changes in prescription size.

**Meaning:**

Early evidence suggests that opioid duration limits had a variable association with postoperative opioid prescribing and should only be part of a larger, multifaceted effort to reduce excessive postoperative opioid prescribing.

## Teaser

This interrupted time series analysis and cross-sectional study investigates the association of opioid prescribing duration limits with postoperative opioid prescribing in Massachusetts and Connecticut.

## Introduction

Excess opioid prescribing for acute pain has come under increased scrutiny in recent years as a major source of unused pills available for misuse and diversion into the community.^1,2,3,4^ Opioid prescribing guidelines published by the Centers for Disease Control and Prevention in March 2016^5^ suggest that more than a 7-day supply of opioids is rarely needed when treating acute pain. However, normative data regarding opioid consumption following surgery are emerging. Many studies suggest that postoperative opioid use may be far less than 7 days, and roughly 80% of pills prescribed remain unused.^6^

To prevent excessive prescribing for acute pain, including postoperative pain, policy efforts have focused on implementing restrictions that limit the duration of opioid prescriptions.^7,8^ Since March 2016, 31 states have implemented legislation to restrict opioid prescribing for acute pain.^9,10^ To date, the association of these limits with prescribing and patient care are unknown. Limiting duration alone still allows for substantial variation in prescribers’ discretion regarding the dosage and number of pills provided in a prescription. For example, a 7-day limit still allows for an initial prescription to range from 42 to 84 tablets of hydrocodone, if the signature is written as 1 to 2 tablets every 4 to 6 hours for 7 days.^3^ Most prescribing limits also have numerous exceptions, with some allowing clinicians to prescribe beyond the limit based on professional medical judgment. Furthermore, these regulations also do not account for heterogeneity in prescribing practices that are driven by clinical and patient need. For instance, a 7-day limit is far beyond what many surgeons would otherwise prescribe for some procedures, suggesting that the limits will have little effect on prescribing.^7^ In other cases, a 7-day limit could be insufficient for the most painful conditions.

Ensuring safe and effective postoperative pain management is critical to ensure high-quality surgical care. In this study, we examined the association of opioid prescribing limits with postoperative opioid prescribing to opioid-naive patients undergoing common surgical procedures in Massachusetts and Connecticut, the first 2 states to implement opioid prescribing limits for acute pain after the Centers for Disease Control and Prevention guidelines were released. We hypothesized that the prescribing limits would not be associated with a significant change in the total amount of opioids prescribed, the duration of initial prescriptions, or the proportion of initial opioid prescriptions exceeding a 7-day supply.

## Methods

### Data Source and Study Cohort

Massachusetts and Connecticut implemented 7-day limits on initial opioid prescriptions on March 14, 2016, and July 1, 2016, respectively. Prescribing limits in both states allow exceptions for patients with cancer-related pain, palliative care needs, and chronic pain management needs; additionally, clinicians may override the limits according to their medical judgment.

Using the 2014 to 2017 IBM MarketScan Research Database (consisting of the IBM MarketScan Commercial Claims and Encounters Database and the Medicare Supplemental and Coordination of Benefits Database), we conducted an interrupted time series analysis and cross-sectional study. We included adults aged 18 years and older who underwent 1 of 24 common surgical procedures between July 1, 2014, and November 30, 2017, in Massachusetts and between March 1, 2015, and November 30, 2017, in Connecticut (procedure codes are listed in eAppendix 1 in the Supplement). We used different starting periods in the 2 states to ensure an equal number of preimplementation and postimplementation data points. We also required that included patients fill at least 1 perioperative opioid prescription (14 days before admission to 3 days after discharge) and that patients were opioid naive at the time of surgery. We limited our study to opioid-naive patients to capture initial opioid prescriptions, the targets of the Massachusetts and Connecticut limits. The limits do not define the term *initial*. Consequently, we followed prior literature and defined opioid naive as the absence of opioid prescriptions between 180 days to 15 days before surgery.^11^

We excluded patients who lacked continuous insurance enrollment from 180 days before admission to 30 days after discharge, patients who had a length of stay exceeding 30 days during the admission for surgery, patients who were not discharged home (eg, died in the hospital), and patients who had subsequent surgical procedures within 30 days of discharge as identified by the occurrence of additional claims with surgical *Current Procedural Terminology* codes. This study was deemed exempt from review and participant informed consent by the University of Michigan institutional review board owing to the use of publicly available deidentified data. This study followed the Strengthening the Reporting of Observational Studies in Epidemiology (STROBE) reporting guideline.

### Outcomes

The primary outcome was the mean prescription size in oral morphine equivalents (OMEs) for the initial postoperative opioid fill. The initial postoperative prescription was defined as the prescription filled closest to surgery between 14 days prior to admission and 3 days after discharge. When more than 1 prescription was filled on the same day, the prescription size was defined as the sum of all prescription sizes filled on that day. When prescriptions were filled an equal number of days before and after surgery, we used the prescription filled after surgery. We used generic and brand drug names to identify opioid medications using National Drug Codes; opioid cough and cold medications were excluded, as were methadone and buprenorphine. We converted prescription amounts into OMEs using the morphine conversion factor for each medication type according to Centers for Disease Control and Prevention documents.^12^ To improve interpretability, results describing opioid amount in OMEs are presented as the equivalent number of pills of 5/325 mg hydrocodone-acetaminophen (1 pill = 5 OMEs).

Secondary outcomes included days supplied in the initial prescription and proportion of initial opioid prescriptions exceeding a 7-day supply. When more than 1 prescription was filled on the same day, the days supplied in the initial prescription was defined as the longest prescription duration filled on that day. We also assessed opioid refills within 30 days of the procedure.

### Covariates

Covariates included age at the time of surgery, sex, tobacco use, Charlson Comorbidity Index score, mental health disorders, and substance use disorders based on *International Classification of Diseases, Ninth Revision, Clinical Modification (ICD-9-CM) *and* International Statistical Classification of Diseases, Tenth Revision, Clinical Modification (ICD-10-CM)* diagnosis codes on claims in the 365 days prior to surgery. Covariates also included whether the surgery was major or minor, classifications that were made based on prior literature.^11,13,14^

The Agency for Healthcare Research and Quality’s Clinical Classification Software was used to create indicators for mental health diagnoses. The subcategories of the mental health diagnoses by the Clinical Classification Software were mood disorders (ie, adjustment, anxiety, and depression), suicide, disruptive behavior disorders (ie, attention-deficit/hyperactivity disorder, conduct and disruptive behavior disorders, and impulse control disorders), personality disorders, schizophrenia, other mental disorders, and substance use disorders (ie, alcohol and other substance-related disorders). Pain disorders included arthritis pain, back pain, neck pain, and other pain disorders.

### Statistical Analysis

We conducted analyses separately for Massachusetts and Connecticut. We used descriptive statistics to compare patient characteristics before and after implementation of limits in each state. To evaluate the association between implementation of the limits and outcomes, we used an interrupted time series approach, a strong quasi-experimental design that tests for level and slope changes in fitted regression lines at the time of interventions. These changes represent potential estimates of the effect of the intervention, assuming that no other events occurred at the time of the intervention that might have affected outcomes and that all other factors are held constant during this period.^15^

For all outcomes except refills, we aggregated outcomes and covariates to the level of months and fit segmented linear regression models to examine immediate level and slope changes in monthly outcomes at the time of implementation. In addition, we examined differences in the slope during the preintervention period and postintervention period to assess for potential changes that could have occurred without the implementation of policy. We assumed that the intervention would result in an abrupt change and created a 1-month washout period between the preintervention and postintervention periods. For Massachusetts, the preimplementation period was defined as months 1 to 20 (July 2014 to February 2016); month 21 was considered a washout period because the limit was implemented in March 2016, and the postimplementation period was months 22 to 41 (April 2016 to November 2017). For Connecticut, the preimplementation period was defined as months 1 to 16 (March 2015 to June 2016); month 17 was considered a washout period because the limit was implemented in July 2016, and the postimplementation period was months 18 to 33 (August 2016 to November 2017). Durbin-Watson statistics were used to evaluate for autocorrelation; any autocorrelation was accounted for in our analyses. For refills, we used a χ^2^ test to compare refill rates before and after limit implementation, as power was limited for this outcome. We considered 2-sided *P* < .05 to be significant. Analyses were conducted using PROC AUTOREG in SAS version 9.4 (SAS Institute).

In our primary analysis, we did not adjust for covariates. To test whether abrupt compositional changes in the sample at the time of implementation might have biased estimates, we conducted a sensitivity analysis in which we controlled for monthly mean age, monthly mean Charlson Comorbidity Index score, monthly percentage female participants, monthly percentage of individuals with tobacco use, monthly percentage of patients with mental health disorders, and monthly percentage of individuals with substance use disorders. In this sensitivity analysis, we also controlled for the monthly percentage of patients undergoing major surgical procedures to assess whether abrupt changes in the types of procedures performed at the time of limit implementation could bias results (eAppendix 2 in the Supplement).

## Results

In total, 16 281 opioid-naive patients (9708 [59.6%] female; median [interquartile range] age range, 45-54 [35-44 to 55-64] years) undergoing surgical procedures were included. In Massachusetts, there were 5340 and 5435 patients in the preimplementation and postimplementation period, respectively. In Connecticut, there were 2869 and 2637 patients in the preimplementation and postimplementation period, respectively. We observed differences between periods with respect to patient characteristics and comorbidities (Table). For example, after prescribing limits were implemented in Massachusetts, we observed a significant increase in tobacco use and mental health disorders, including adjustment disorder, anxiety, depression, and substance use. After limit implementation in Connecticut, patients had a significant increase in Charlson Comorbidity Index score, anxiety, and substance use. Overall, the magnitude of these differences was small.

**Table.** Demographic and Clinical Attributes of the Study Cohort Before and After Opioid Prescribing Policy Limits

Characteristic	Patients in Massachusetts, No. (%)		*P* Value	Patients in Connecticut, No. (%)		*P* Value
	Before Limits (n = 5340)	After Limits (n = 5435)		Before Limits (n = 2869)	After Limits (n = 2637)	
Age, y						
≤34	1003 (18.8)	982 (18.1)	<.001	491 (17.1)	445 (16.9)	<.001
35-44	1005 (18.8)	1077 (19.8)		466 (16.2)	426 (16.2)	
45-54	1332 (24.9)	1392 (25.6)		783 (27.3)	755 (28.6)	
55-64	1468 (27.5)	1580 (29.1)		901 (31.4)	886 (33.6)	
65-74	370 (6.9)	266 (4.9)		137 (4.8)	88 (3.3)	
≥75	162 (3.0)	138 (2.5)		91 (3.2)	37 (1.4)	
Sex						
Male	2045 (38.3)	2103 (38.7)	.67	1256 (43.8)	1169 (44.3)	.68
Female	3295 (61.7)	3332 (61.3)		1613 (56.2)	1468 (55.7)	
Tobacco use	268 (5.0)	346 (6.4)	.003	208 (7.2)	221 (8.4)	.12
Comorbidities						
None: CCI score 0	3459 (64.8)	3478 (64.0)	.36	1777 (61.9)	1658 (62.9)	.01
Mild: CCI score 1-2	1446 (27.1)	1505 (27.7)		863 (30.1)	746 (28.3)	
Moderate: CCI score 3-4	301 (5.6)	290 (5.3)		178 (6.2)	153 (5.8)	
Severe: CCI score ≥5	134 (2.5)	162 (3.0)		51 (1.8)	80 (3.0)	
Mental health disorders						
Adjustment	240 (4.5)	302 (5.6)	.01	89 (3.1)	96 (3.6)	.27
Anxiety	606 (11.3)	801 (14.7)	<.001	323 (11.3)	343 (13.0)	.05
Depression	650 (12.2)	739 (13.6)	.03	277 (9.7)	236 (8.9)	.37
Suicide	7 (0.1)	12 (0.2)	.27	2 (0.1)	5 (0.2)	.21
Personality	10 (0.2)	13 (0.2)	.56	5 (0.2)	5 (0.2)	.89
Disruptive	100 (1.9)	142 (2.6)	.01	45 (1.6)	44 (1.7)	.77
Schizophrenia	19 (0.4)	14 (0.3)	.36	7 (0.2)	5 (0.2)	.67
Other mental	209 (3.9)	327 (6.0)	<.001	91 (3.2)	106 (4.0)	.09
Drug and substance use	136 (2.5)	315 (5.8)	<.001	87 (3.0)	158 (6.0)	<.001
Pain disorders						
Arthritis	2437 (45.6)	2509 (46.2)	.58	1317 (45.9)	1175 (44.6)	.36
Back	1101 (20.6)	1174 (21.6)	.21	601 (20.9)	560 (21.2)	.79
Neck	465 (8.7)	549 (10.1)	.01	273 (9.5)	266 (10.1)	.48
Other pain	1124 (21.0)	1332 (24.5)	<.001	626 (21.8)	581 (22.0)	.85

Abbreviation: CCI, Charlson Comorbidity Index.

### Massachusetts

**Figure 1.**
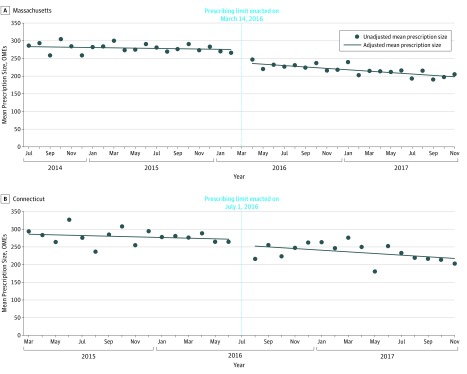
Mean Size of Initial Postoperative Prescription Before and After Opioid Legislation in Massachusetts and Connecticut A, In Massachusetts, implementation of the limit was associated with an immediate level decrease in prescription size (−38 oral morphine equivalents [OMEs] [95% CI, −44 to −32 OMEs]) and slope decrease (−1.5 OMEs/mo [95% CI −2.1 to −0.9 OMEs/mo]). B, In Connecticut, implementation of the limit was not associated with a significant immediate level change in prescription size (−18 OMEs [95% CI, −49 to 14 OMEs]) or slope change (−1.3 OMEs/mo [95% CI, −4.6 to 2.0 OMEs/mo]).

**Figure 2.**
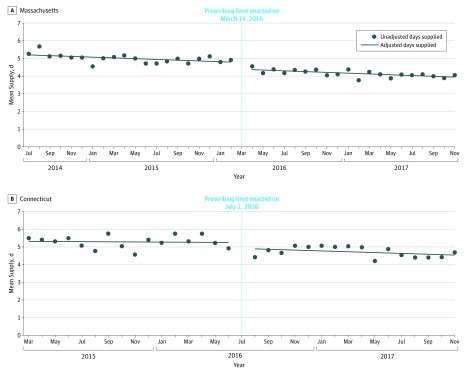
Mean Days Supplied in the Initial Postoperative Prescription Before and After Opioid Legislation in Massachusetts and Connecticut A, In Massachusetts, implementation of the limit was associated with an immediate level decrease in days supplied (−0.4 days [95% CI, −0.6 to −0.2 days]) but no significant slope change (0 d/mo [95% CI, −0.02 to 0.02 d/mo]). B, In Connecticut, implementation of the limit was not associated with a significant immediate level change in days supplied (−0.3 days [95% CI, −0.7 to 0.1 days]) or slope change (0 d/mo [95% CI, 0-0 d/mo]).

**Figure 3.**
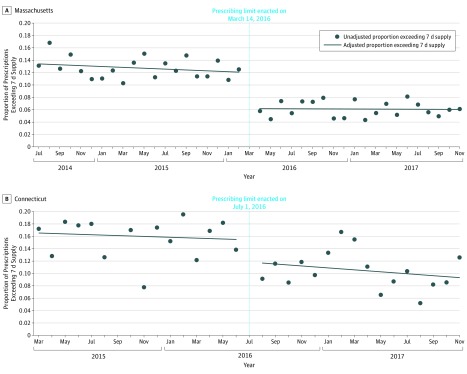
Proportion of Prescriptions Exceeding a 7-Day Supply Before and After Opioid Legislation in Massachusetts and Connecticut A, In Massachusetts, implementation of the limit was associated with an immediate level decrease in the proportion of prescriptions exceeding a 7-day supply (−5.9 percentage points [95% CI, −7.9 to −3.9 percentage points]) but without a significant slope change (0.1 percentage points [95% CI, −0.1 to 0.3 percentage points]). B, In Connecticut, implementation of the limit was not associated with an immediate level change in the proportion of prescriptions exceeding a 7-day supply (−3.7 percentage points [95% CI, −8.2 to 0.8 percentage points]) or slope change (0.1 percentage points [95% CI, −0.7 to 0.5 percentage points]).

In July 2014, prior to limit implementation, patients in Massachusetts filled a mean of 284 OMEs (56.8 pills) in the initial postoperative opioid prescription. Opioid prescriptions were supplied for a mean of 5.2 days, and 13.5% of prescriptions exceeded a 7-day supply. Implementation of the limit was associated with an immediate mean level decrease in prescription size (−38 OMEs or 7.6 pills [95% CI, −44 to −32 OMEs]) and a mean slope decrease (−1.5 OMEs/mo [95% CI, −2.1 to −0.9 OMEs/mo]) (Figure 1A). Implementation of the limit was associated with an immediate mean level decrease in days supplied (−0.4 days [95% CI, −0.6 to −0.2 days]) but not a significant slope change (0 d/mo [95% CI, −0.02 to 0.02 d/mo]) (Figure 2A). Implementation of the limit was associated with an immediate mean level decrease in the proportion of prescriptions exceeding a 7-day supply (−5.9 percentage points [95% CI, −7.9 to −3.9 percentage points]) but not a slope change (0.1 percentage points/mo [95% CI, −0.1 to 0.3 percentage points/mo]) (Figure 3A). The refill rate within 30 days was not different before (13.8%) and after (13.0%) limit implementation. Conclusions were unchanged in our sensitivity analysis in Massachusetts when additionally controlling for covariates and the percentage of patients undergoing major surgery.

### Connecticut

In March 2015, prior to limit implementation, patients in Connecticut filled a mean of 288 OMEs (57.6 pills) in the initial postoperative opioid prescription. Opioid prescriptions were supplied for a mean of 5.3 days, and 16.6% of prescriptions exceeded a 7-day supply. Implementation of the limit was not associated with an immediate mean level change in prescription size (−18 OMEs [95% CI, −49 to 14 OMEs]) or slope change (−1.3 OMEs/mo [95% CI, −4.6 to 2.0 OMEs/mo]) (Figure 1B). Implementation of the limit was also not associated with an immediate level change in days supplied (−0.3 days [95% CI, −0.7 to 0.1 days]) or slope change (0 d/mo [95% CI, 0 to 0 d/mo]) (Figure 2B). In addition, implementation of the limit was not associated with an immediate mean level change in the proportion of opioid prescriptions exceeding a 7-day supply (−3.7 percentage points [95% CI, −8.2 to 0.8 percentage points]) or slope change (−0.1 percentage points/mo [95% CI, −0.7 to 0.5 percentage points/mo]) (Figure 3B). Similar to Massachusetts, we did not observe significant changes in prescription refills following the implementation of prescribing limits. The refill rate within 30 days was not different before (13.0%) and after (13.2%) limit implementation. Conclusions were unchanged in our sensitivity analysis in Connecticut when additionally controlling for covariates and the percentage of patients undergoing major surgery.

## Discussion

Early evidence suggests that implementation of opioid duration limits had variable association with postoperative opioid prescribing in Massachusetts and Connecticut. The mean opioid prescription size filled, duration supplied, and prescribing exceeding a 7-day supply decreased after limit implementation in Massachusetts, but not in Connecticut. However, even in Massachusetts, the magnitude of the observed changes was small, and the amount of opioid prescribed still exceeded the mean amount used by opioid-naive patients during recovery for many procedures.^16,17,18^ Our findings suggest that opioid prescribing limits for acute pain may have modest ability to reduce excessive postoperative opioid prescribing.

The reasons for the lack of substantial change after limit implementation are likely multifactorial. First, as noted previously, a 7-day limit could represent as many as 84 tablets of 5 mg of hydrocodone (420 OMEs) for a prescription of 2 tablets every 4 hours, a level that far exceeded the mean amount prescribed before policy implementation in both states. Consequently, there were very few prescriptions that could be affected by the limits. Second, the limits allowed for exceptions based on professional judgment. It is possible that prescribers are using this exception to avoid the perceived risk of lower patient satisfaction, uncontrolled pain, or prescription refill.^19,20^ This is further supported by the significant, albeit small, increase in related risk factors for pain- and opioid-related outcomes, including substance use and anxiety, after prescribing limits were implemented in both states. Furthermore, the prescribing limits are designed in a way that could be circumvented by practitioners.^7^ Anecdotal reports suggest that some physicians are prescribing multiple small prescriptions but postdating subsequent prescriptions, thereby providing durations of therapy beyond the limit while still technically being compliant with the limits.^21^

The modest findings in our study differ somewhat from prior studies that have shown greater reductions in opioid prescribing. In 2016, Rhode Island implemented limits to restrict opioid prescribing by the total number of doses and the OMEs prescribed per day, and not by the days supplied. These limits reduced opioid prescription by more than 50% after lumbar spine surgery at a single institution.^22^ Another single-institution study in Florida observed a decrease by more than 30% in opioid prescriptions for patients undergoing common outpatient surgical procedures after prescribing limit implementation.^23^ It should be noted that Rhode Island’s limit is much more restrictive in that it limits opioid prescriptions to 20 doses, while Florida’s limit restricts prescriptions to a 3-day supply. Even if some variation on limits may be able to achieve greater prescribing reductions, it is important to consider that the goal is not absolute reduction alone, but rather reductions of prescribing that exceed clinical need. As such, prescribing restrictions based on number of days alone or number of doses may miss important nuances of perioperative care, such as prior opioid exposure, procedure type, and patient-level risk factors.^24^

An alternative approach to reduce excessive prescribing may be to develop and implement evidence-based prescribing guidelines based on patient-reported opioid-related outcomes, including opioid consumption, use of opioid alternatives, pain, and satisfaction.^3,16,17,18^ For example, Hill et al^17^ developed opioid prescribing guidelines based on the amount consumed while being an inpatient to direct the amount prescribed for outpatient pain control after surgery. This approach was able to effectively tailor postoperative opioid prescribing by accounting for patient variability. Evidence-based prescribing guidelines have also proven to be effective in reducing opioid prescribing after surgery without increasing the need for refills in multiple other contexts.^3,16,17,18^

### Limitations

Our study has several limitations. First, it only captures opioid prescriptions reimbursed through employer-based health insurance and did not allow us to examine trends among individuals covered by other mechanisms or filling opioid prescriptions outside of insurance or individuals who were uninsured. Second, we did not examine all surgical procedures performed in Massachusetts and Connecticut, although the inclusion of patients across a broad variety of procedures and institutions is a strength compared with prior studies that have focused on single institutions or procedures. Third, we explored the possibility of using other states that did not implement a policy limit during the study period as a control group (eg, Wisconsin). However, we could not identify states that had parallel preintervention trends for all 3 outcomes, likely owing to other contextual factors. Our review of the policies implemented in these 2 states did not identify any other events occurring at the same time of implementation that might have biased results. Fourth, we could not evaluate downstream outcomes such as longer-term refills or return visits for pain owing to limited power. Additional studies will be needed to further understand the long-term impact of current opioid prescribing limits and clinical appropriateness of any observed reductions. Fifth, our sample sizes for Connecticut were limited, potentially resulting in a lack of power. However, even if greater power would have resulted in statistical significance, it should be noted that the effect sizes in Connecticut were small, as they were in Massachusetts.

## Conclusions

The results of this interrupted time series analysis and cross-sectional study suggest that prescribing limits have variable association with postoperative opioid prescribing and should only be part of a larger, multifaceted effort to reduce excessive postoperative opioid prescribing. Legislative approaches should be coupled with evidence-based, patient-centered guidelines to yield appropriate and safe postoperative pain management.

## Notes

Supplement. **eAppendix 1.** Study Cohort Surgery Classification **eAppendix 2.** Sensitivity Analyses Adjusting for Clinical Characteristics and Case Mix
