# Supplementary material for: Association of State Opioid Duration Limits With Postoperative Opioid Prescribing
Source: JAMA Netw Open. 2019 Dec 27;2(12):e1918361. doi: 10.1001/jamanetworkopen.2019.18361 (PMC6991309; doi:10.1001/jamanetworkopen.2019.18361)
Supplement: Supplement. — eAppendix 1. Study Cohort Surgery Classification eAppendix 2. Sensitivity Analyses Adjusting for Clinical Characteristics and Case Mix [file jamanetwopen-2-e1918361-s001.pdf]

Supplementary Online Content

Agarwal S, Bryan JD, Hu HM, et al. Association of state opioid duration limits with postoperative opioid prescribing. *JAMA Netw Open*. 2019;2(12):e1918361. doi:10.1001/jamanetworkopen.2019.18361

eAppendix 1. Study Cohort Surgery Classification

eAppendix 2. Sensitivity Analyses Adjusting for Clinical Characteristics and Case Mix

This supplementary material has been provided by the authors to give readers additional information about their work.

© 2019 Agarwal S et al. *JAMA Network Open.*

eAppendix 1. Study Cohort Surgery Classification

Minor Surgery (CPT-4 code)	Major Surgery (CPT-4 Code)
Neuroplasty Transposition (64702, 64704, 64708, 64712-64714, 64716, 64718-64719, 64721)	Heart Valve Procedure(s) (33361-33369, 33390-33391, 33400-33401, 33403, 33405-33406, 33410-33413, 33420, 33422, 33425-33427, 33430, 33460, 33463-33465, 33468, 33470-33471, 33474-33475)
Thyroidectomy (60210, 60212, 60220, 60225, 60240, 60252, 60254, 60260, 60270-60271)	Coronary Artery Bypass Graft (33510-33514, 33516-33519, 33521-33523, 33530, 33533-33536)
Appendectomy (44950, 44955, 44960, 44970)	Endarterectomy (35301-35306, 35311, 35321, 35331, 35341, 35351, 35355, 35361, 35363, 35371-35372)
Hemorrhoid Procedure (46221, 46250, 46255, 46257-46258, 46260-46262, 46320)	Small Bowel Resection and/or Enterolysis (44120-44121, 44125-44128, 44180)
Cholecystectomy (47600, 47605, 47610, 47612, 47620, 47562-47564)	Colectomy, Proctectomy (44140-44141, 44143-44147, 44150-44151, 44155-44158, 44160, 44204-44208, 44210-44213, 45110-45114, 45116, 45119-45121, 45123)
Inguinal Femoral Hernia Repair (49505, 49507, 49520-49521, 49525, 49550, 49553, 49555, 49557, 49560-49561, 49565-49566, 49568, 49570, 49572, 49585, 49587, 49590, 49650-49657)	Nephrectomy (50220, 50225, 50230, 50234, 50236, 50240, 50320, 50543-50548)
Lumpectomy and/or Mastectomy (19300-19307)	Lap and Open Prostatectomy (55801, 55810, 55812, 55815, 55821, 55831, 55840, 55842, 55845, 55866)
Debridement of Wound (11000-11001, 11004-11006, 11008, 11010-11012, 11042-11047)	Hysterectomy (58150, 58152, 58180, 58200, 58210, 58260, 58262-58263, 58267, 58270, 58275, 58280, 58285, 58290-58294, 58570-58573, 58541-58546, 58548, 58550, 58552-58554, 58661)
Excision of Skin Lesion(s) (11400-11404, 11406, 11420-11424, 11426, 11440-11444, 11446, 11600-11604, 11606, 11620-11624, 11626, 11640-11644, 11646)	Cesarean Section (59514-59515)
	Total Hip Arthroplasty (27125, 27130, 27132, 27134, 27137-27138)
	Total Knee Arthroplasty (27437-27438, 27440-27443, 27445-27447)
	Skin Graft(s) (15050, 15100-15101, 15110-15111, 15115-15116, 15130-15131, 15135-15136, 15150-15152, 15155-15157, 15200-

© 2019 Agarwal S et al. *JAMA Network Open.*

	15201, 15220-15221, 15240-15241, 15260-15261)
	Gastric Bypass, Gastrectomy (43620-43622, 43631-43634, 43644-43645, 43775, 43842-43843, 43845-43847, 43886-43888)
	Abdominal Aortic Aneurysm Repair (33860, 33863-33864, 33870, 33875, 33877, 34800, 34802-34805)
	Laminectomy (63001, 63003, 63005, 63011-63012, 63015-63017, 63020, 63030, 63035, 63040, 63042-63048, 63050-63051, 63055-63057, 63064, 63066, 63075-63078, 63081-63082, 63085-63088, 63090-63091, 63101-63103, 63170, 63172-63173, 63180, 63182, 63185, 63190-63191, 63194-63200, 63250-63252, 63265-63268, 63270-63273, 63275-63278, 63280-63283, 63285-63287, 63290, 63295, 63300-63308, 63655)

© 2019 Agarwal S et al. *JAMA Network Open.*

eAppendix 2. Sensitivity analyses adjusting for clinical characteristics and case mix.

	Massachusetts (Sensitivity Analyses)		Massachusetts (Main Results)		Connecticut (Sensitivity Analyses)		Connecticut (Main Results)	
	**Coefficient**	**95% CI**	**Coefficient**	**95% CI**	**Coefficient**	**95% CI**	**Coefficient**	**95% CI**
**Mean Prescription Size (OME)**								
Intercept	203	[114, 292]	284	[280, 289]	245	[93, 397]	287	[264, 311]
Pre-intervention slope	-0.1	[-0.7, 0.5]	-0.4	[-0.8, 0.0]	-2.5	[-5.8, 0.8]	-1.0	[-3.4, 1.4]
Level change	**-38**	**[-49, -27]**	**-38**	**[-45, -32]**	-22	[-62, 17]	-17.5	[-49.3, 14.3]
Slope Change	**-2.3**	**[-3.3, -1.3]**	**-1.5**	**[-2.1, -0.9]**	0.5	[-3.6, 4.6]	-1.3	[-4.6, 2.0]
**Days Supplied**								
Intercept	4.7	[2.9, 6.5]	5.2	[5.1, 5.4]	6.8	[4.6, 9.0]	5.3	[4.9, 5.7]
Pre-intervention slope	-0.03	[-0.05, -0.01]	-0.02	[-0.04, 0.00]	0.0	[0.0, 0.0]	0.0	[0.0, 0.0]
Level change	**-0.4**	**[-0.7, -0.2]**	**-0.4**	**[-0.6, -0.2]**	-0.4	[-1.0, 0.2]	-0.3	[-0.7, 0.1]
Slope Change	0.0	[-0.02, 0.02]	0.0	[-0.02, 0.02]	0.0	[0.0, 0.0]	0.0	[0.0, 0.0]
**Proportion of Prescriptions Exceeding a 7-day Supply (%)**								
Intercept	4.4	[-10.7, 19.5]	13.5	[12.1, 14.9]	31.1	[11.1, 51.1]	16.6	[13.3, 19.9]
Pre-intervention slope	-0.1	[-0.3, 0.1]	-0.1	[-0.3, 0.1]	-0.1	[-0.5, 0.3]	-0.1	[-0.5, 0.3]
Level change	**-5.7**	**[-7.9, -3.5]**	**-5.9**	**[-7.9, -3.9]**	-3.4	[-8.7, 1.9]	-3.7	[-8.2, 0.8]
Slope Change	0.1	[-0.1, 0.3]	0.1	[-0.1, 0.3]	0.1	[-0.5, 0.7]	-0.1	[-0.7, 0.5]

Adjusting for clinical characteristics and case mix did not result in substantive changes in conclusions, suggesting that estimates were not biased by abrupt changes in these covariates at the time of implementation.

© 2019 Agarwal S et al. *JAMA Network Open.*
